# Successful treatment of severe venous leg ulcers and diabetic foot ulcers using ozone

**DOI:** 10.1016/j.jvsv.2025.102278

**Published:** 2025-06-13

**Authors:** Giuseppe Masiello, Marianno Franzini, Umberto Tirelli, Tommaso Richelmi, Luigi Valdenassi, Francesco Vaiano, Salvatore Chirumbolo

**Affiliations:** a Division of Antalgic Therapy and Rehabilitation, Clinics Salus Fisiocenter, Capurso, Bari, Italy; b Italian Scientific Society of Oxygen-Ozone Therapy (SIOOT) and High Master School of Oxygen-Ozone Therapy, University of Pavia, Pavia, Italy; c Tirelli Medical Group, Pordenone and Former Head of the Oncology Unit, National Cancer Institute, Aviano, Pordenone, Italy; d Department of Engineering for Innovation Therapy, University of Verona, Verona, Italy

**Keywords:** Ozone therapy, Ulcers, Venous, Diabetic foot, Healing, Antibiotics

## Abstract

**Background:**

Venous leg ulcers (VLUs) and diabetic foot ulcers (DFUs) are chronic wounds associated with significant morbidity, high recurrence rates, and poor healing outcomes. Conventional treatments often fail to achieve satisfactory results, leading to prolonged pain, infection risks, and reduced quality of life. Oxygen-ozone therapy (OOT) has emerged as a potential adjunct to conventional wound care, with antimicrobial, anti-inflammatory, and tissue-regenerating properties. This study evaluates the efficacy of OOT in treating severe VLUs and DFUs.

**Methods:**

A total of 25 patients (mean age, 57.2 ± 10.51) with refractory VLUs (n = 18) or DFUs (n = 7) received OOT alongside standard care. Treatment protocols included major autohemotherapy (O_2_-O_3_-MAHT), topical ozone application, and localized ozone injections. Clinical assessments included pain scoring (Numeric Rating Scale), microbiological evaluations, and logistic regression analysis to determine healing rates.

**Results:**

At 4 weeks, pain scores decreased by 73.27% (*P* < .0001), and 92% of septic lesions showed negative bacterial cultures. Logistic regression analysis indicated a significant improvement in healing rates (*P* < .0001), with 80% of patients achieving complete wound closure. Inflammatory markers were reduced, and tissue regeneration was enhanced.

**Conclusions:**

OOT demonstrated substantial efficacy in reducing infection, alleviating pain, and promoting wound healing in patients with severe VLUs and DFUs, restoring their healthy, normal limbs. These findings support the use of OOT as a valuable adjunctive treatment. Further large-scale, randomized trials are needed to optimize treatment protocols and confirm long-term benefits.

**Article Highlights**

• **Type of Research:** Experimental research
• **Key Findings:** The patient cohort was 25 patients with severe venous leg ulcers (VLUs) and diabetic foot ulcers (DFUs), unresponsive to conventional treatments. The treatment approach was a combination of standard care and oxygen-ozone therapy, including major autohemotherapy, topical ozone applications, and ozone injections. In terms of efficacy, 80% of patients achieved complete wound healing. Pain scores decreased by 73.27% after 4 weeks. Ninety-two percent of septic wounds had negative bacterial cultures after treatment. Inflammatory biomarkers (C-reactive protein, erythrocyte sedimentation rate, interleukin-6) significantly reduced. No major adverse events or amputations were reported.
• **Take Home Message:** Oxygen-ozone therapy, when used as an adjunct to standard care, shows significant promise in enhancing wound healing, reducing infection and inflammation, and alleviating pain in chronic venous leg ulcers and diabetic foot ulcers. This approach offers a viable, noninvasive alternative for patients with treatment-resistant ulcers.

Venous leg ulcers (VLUs) represent a major concern in the clinical management of chronic venous insufficiency and venous hypertension,^1^^,^^2^ classically appearing on the lower limb and including from 60% to 80% of all leg ulcerations, according to recent data,^3^^,^^4^ involving at least 1.69% of the United States (U.S.) elderly population.^5^ Noticeably, according to recent evidence, at least three different studies have reported VLUs prevalence ranging from 0.12% to 1.69% and an incidence ranging from 0.3% to 1.33%.^6^, ^7^, ^8^, ^9^ VLU prevalence in Europe is comparable to global surveys, generally affecting 1% to 2% of the population, with higher rates in the elderly, whereas in Italy there is a prevalence of approximately 1.5% in the general population, increasing to 4% to 5% in those over 65 years old.^10^^,^^11^ The increase in the rate of VLUs occurrence in the elderly represents a paramount matter of debate for clinics, due to the ever-increasing aged population in Western countries and being associated with an ongoing declining in the birth rate and an amelioration in the current life expectancies. VLUs are open lesions in the lower limbs with the concerning issue of opportunistic infections, sepsis, and even antibiotic resistance.^12^, ^13^, ^14^, ^15^ This concerning issue includes also diabetic foot ulcers (DFUs), which, although differentially described from VLUs, represent altogether a major challenge for therapy of ulcerous wounds and their prevention from microbial infections.^16^ Conventional therapy for VLUs focuses on comprehensive management through compression therapy, effective wound care, appropriate pharmacotherapy, and lifestyle modifications. Patient education and regular follow-up are essential to ensure adherence to treatment and prevent recurrence. This multifaceted approach aims to improve healing outcomes and enhance the quality of life for individuals severely affected by VLUs.^17^, ^18^, ^19^ Usually, healing rates are reliable in only 60% of patients by 12 weeks from starting therapy, and often, once healed, at least 75% develop a concerning recurrence within 3 weeks with severe pain and disability.^20^^,^^21^ As VLUs are predominant in older adults, often with the concurrence of chronic venous insufficiency, their exacerbation, due to therapy negligence, pharmacological failure or malpractice, along with a comprehensible weakness in the immune defense due to senescence, might finally lead to limb amputations.^22^

### Preventing this occurrence is of utmost importance

Ozone showed paramount effects on chronic non-healing wounds, such as severe VLUs or DFUs.^23^, ^24^, ^25^ The ability of ozone to counteract bacterial infection and growth^26^ and promote wound healing^27^^,^^28^ suggested us to use ozone therapy, following protocols from the Italian Scientific Society of Oxygen-Ozone Therapy (SIOOT) with updates^29^ for a cohort of lower limb severe ulcerous wounds, with failure in conventional therapy and/or recurrence. Results are discussed below.

## Methods

### Subjects’ enrollment

A total number of 25 patients (mean age, 57.2 ± 10.51 years), 16 females and nine males, from an initially enrolled number of 27 patients, were involved in the study, as two patients withdrew from the study before completing it for personal issues. Sample size evaluation at 95% confidence interval (CI), reported that 25 samples ensured a 95% chance that the real value is within ±4.76% (<5.00%) of the evaluated data, involving 98.5% of the population proportion.

### Eligibility criteria

Eligibility criteria included patients presenting with painful, severe manifestations of VLUs or DFUs refractory to conventional therapy, often associated with ambulation disability and a history of recurrence or poor prognosis. All patients underwent a standardized vascular assessment before inclusion.

• Arterial circulation was evaluated using ankle-brachial index (ABI) measurements and duplex ultrasound. Patients with critical limb ischemia (ABI <0.5) or requiring immediate revascularization were excluded. Minor degrees of arterial calcification were accepted if macrovascular perfusion was preserved (ABI, 0.7-1.3).
• Patients with heavy arterial calcification affecting the reliability of ABI measurements were assessed with toe-brachial index when necessary.
• Venous insufficiency was assessed via duplex ultrasound of the lower limbs. Presence of superficial or deep venous reflux was documented. Patients with significant superficial venous reflux were eligible provided that it was managed before inclusion, either via compression therapy or surgical/endovenous intervention. Those with deep venous reflux were carefully reviewed, and only patients with compensated deep reflux without active venous obstruction were included.

### Exclusion criteria

Exclusion criteria included: uncontrolled severe arterial disease (requiring revascularization), non-compliance with vascular compression, active deep vein thrombosis, glucose-6 phosphate dehydrogenase deficiency (favism), pregnancy, severe hyperthyroidism, malignancy, psychiatric disorders, epilepsy, and intravenous drug users.

Prior to enrolment, all patients underwent surgical debridement and optimization of underlying vascular conditions when indicated. The procedure involved manual sharp debridement using sterile scalpels and curettes to remove necrotic tissue and biofilm, exposing healthy tissue. This was conducted by experienced wound care specialists under sterile conditions and in accordance with established outpatient wound care protocols. For non-septic lesions, pharmacological treatment included supportive therapies such as antiplatelet agents, vasodilators, antihypertensives, antidiabetics, and nutritional support where appropriate. No systemic antibiotics were used unless clinical signs of infection emerged. This conservative approach was integrated with oxygen-ozone therapy (OOT) to promote healing and prevent secondary infections.

Patients continued their standard pharmacologic therapies without interruption during the adjunct OOT. All patients signed an informed consent before entering the study. As outpatients referring to the clinical health care service for being treated with OOT, the study underwent disclaiming for a mandatory approval by the local ethical committee and is in compliance with the Helsinki Declaration. Patients exhibited ulcerous wounds, either from diabetes or venous ulcers, in the lower limbs for 6 months (n = 20) or 1 year (n = 5), and were treated with a one single antibiotic treatment (n = 15) or multiple antibiotic treatments (n = 10), which included amoxicillin-clavulanate (n = 9), cephalexin (n = 4), clindamycin (n = 3), ceftriaxone (n = 17), and daptomycin (n = 3). No patient had antibiotic-resistant bacteria. Before treatment, five patients experienced previous events of myocardial acute infarction, 10 of atrial fibrillation, five of occlusive arterial pathology of lower limbs, seven had type 2 diabetes, 18 had VLUs and seven had DFUs, of which 15 had infected wounds and 10 were without infection or sepsis. Patients undergoing pharmacological treatments for these ailments did not interrupt therapy upon adjunct OOT. Although DFUs and VLUs are distinct pathophysiological entities, they share common characteristics such as chronicity, impaired wound healing, high risk of infection, and significant morbidity. In this study, both types of chronic ulcers were included to broadly assess the effectiveness of adjunctive OOT in promoting wound healing across different etiologies of chronic lower limb wounds. It is important to note that revascularization procedures were not mandated prior to enrollment. Patients were managed according to standard clinical practices, which included ongoing pharmacologic therapy and compression therapy where appropriate. The inclusion of patients who were refractory to conventional therapies allowed us to specifically evaluate the potential role of OOT in difficult-to-heal wounds, regardless of the underlying vascular status, provided that no critical limb ischemia or need for immediate surgical revascularization was present.

### Wound evaluation and assessment protocol

This study was a prospective observational cohort study designed to assess the effects of adjunctive OOT on the healing of severe VLUs and DFUs. Patients were enrolled consecutively, consented prior to participation, and treated following a standardized protocol without randomization or comparison to a placebo group. Clinical outcomes, including pain levels, wound healing, microbiological status, and inflammatory biomarkers, were prospectively collected over a 12-week period, following routine clinical practice enhanced by the adjunct OOT. Wound evaluation was performed according to previously reported guidelines,^30^, ^31^, ^32^, ^33^ systematically at baseline and then weekly until the end of the 4-week treatment period. Assessments followed internationally validated guidelines for chronic wound monitoring, including clinical wound evaluation, wound measurement, and standardized photographic documentation. For each wound, the following parameters were recorded: (1) Wound area (cm^2^) was measured using a transparent sterile grid and planimetry. The largest length and width were recorded with a sterile ruler and calculated as length × width for approximate surface estimation. (2) Tissue type was categorized as necrotic, slough, granulation, or epithelial tissue, following standard definitions. (3) Exudate amount was graded as none, light, moderate, or heavy. (4) Exudate type was categorized as serous, serosanguinous, purulent, or bloody. (5) Wound edge and periwound skin were assessed for maceration, induration, erythema, and signs of infection. (6) Presence of odor was also recorded.

Although specific area measurements were not systematically recorded for all patients, the ulcers treated in this study were classified as severe based on clinical presentation, chronicity (≥6 months), and refractoriness to conventional therapy. The average wound size, measured in a representative subgroup of 12 patients, ranged from 6.5 cm^2^ to 18.2 cm^2^, with a mean area of approximately 12.1 ± 3.9 cm^2^. These values align with what is typically classified in the literature as clinically significant chronic ulcers requiring advanced intervention.

**Supplementary Figure 1:**
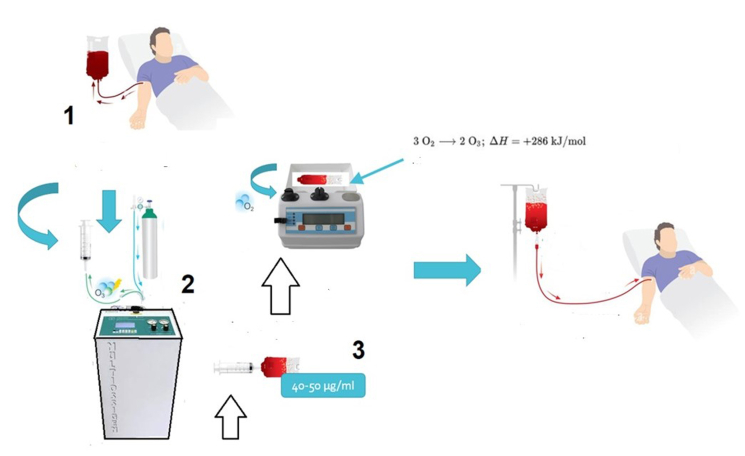
Numeric Rating Scale (NRS) questionnaire with 10-score points to evaluate pain and discomfort in the patient; relative scores are reported.

Pain associated with the wound was assessed by the patient using the Numeric Rating Scale (NRS), (Supplementary Fig S1, online only) with independent confirmation by two interviewers (see below). Photography was performed under consistent conditions (fixed light, distance, and angle) at each assessment point using a standardized imaging protocol. Before starting OOT, surgical debridement was performed in all patients to remove devitalized tissue, as recommended by international wound care guidelines. The protocol for wound evaluation was based on the World Union of Wound Healing Societies (WUWHS) guidelines and consistent with previous validated studies. In patients with non-septic wounds, pharmacological support included ongoing medications for comorbidities (eg, diabetes, vascular insufficiency), such as oral antidiabetics, antihypertensives, and antiplatelet agents, alongside nutritional supplementation and wound care. No systemic antibiotics were administered unless signs of infection developed during the study. Treatment protocols were tailored according to lesion type, with distinct approaches for septic and non-septic ulcers.

### Vascular assessment results

Among the included patients, five had a documented history of peripheral arterial occlusive disease but with ABI >0.7 following intervention or conservative management. Venous duplex ultrasound demonstrated venous reflux in 16 patients: 10 had superficial reflux, whereas six had deep venous reflux. In the superficial reflux group, eight patients underwent vein ablation procedures prior to study inclusion, whereas two were managed with compression therapy alone. No interventions were performed on deep venous reflux; these patients were managed conservatively with compression.

### Clinical biochemistry and hematology assays

Routine hematologic and inflammatory markers were assessed at baseline (week 0), mid-treatment (week 4), and study completion (week 10) to monitor systemic inflammation and response to therapy. These included: (1) erythrocyte sedimentation rate (ESR), measured using the Westergren method. Blood samples were anticoagulated with sodium citrate, and sedimentation was measured in millimeters after 1 hour. ESR was used as a nonspecific marker of inflammation and tissue damage. (2) C-reactive protein (CRP) was quantified using high-sensitivity immunoturbidimetric assay on an automated analyzer (Roche Cobas). CRP levels reflect acute-phase response to infection or inflammation and were reported in mg/L; (3) Interleukin-6 (IL-6), measured using an enzyme-linked immunosorbent assay (ELISA), with commercially available high-sensitivity kits (R&D Systems). IL-6 concentrations were expressed in pg/mL and were used to evaluate the pro-inflammatory cytokine profile of patients. All blood samples were collected in the morning after fasting, processed within 2 hours, and analyzed in certified clinical laboratories according to standard operating procedures and manufacturer guidelines.

### Microbiological evaluation of lesions

Cutaneous swabs for microbiological assay were performed for any lesion, according to previous reports,^32^ following standard microbiological techniques with McConkey agar (prevalent), mannitol salt agar, and blood agar (when requested). Positivity was attained with presence of colonies ≥10^5^ colony-forming units (CFU)/mL. Cases with positive cultures but no signs of inflammation were not classified as septic but colonized.

Surgical debridement was carried out in the outpatient clinic using sterile scalpels and curettes under aseptic conditions. This procedure aimed to remove devitalized tissue, slough, and biofilm, ensuring better exposure of the wound bed to topical ozone therapy and enhancing sampling reliability for microbiological evaluation.

Cutaneous swabs for microbiological assay were performed for each lesion following surgical debridement to remove necrotic tissue and superficial contaminants. The debridement was carried out using sterile surgical instruments under aseptic conditions, ensuring exposure of viable wound bed tissue. This step was crucial to avoid confounding by superficial microbial colonization and allowed accurate assessment of infectious agents within the wound. Swabs were collected using standard microbiological techniques and cultured on McConkey agar (mainly), mannitol salt agar, and blood agar (as needed). As highlighted before, the result was considered positive with the presence of bacterial colonies ≥10^5^ CFU/mL. Although primary pathogen identification was conducted, antimicrobial susceptibility testing (AST) using standard disk diffusion (Kirby-Bauer method) was performed selectively on isolates to assess resistance profiles. Based on AST results, none of the bacterial isolates demonstrated significant resistance to first-line antibiotics, confirming the appropriateness of empirical antibiotic regimens (including amoxicillin-clavulanate, cephalexin, clindamycin, ceftriaxone, and daptomycin).

However, we acknowledge that systematic AST was not performed for all isolates, and therefore some low-frequency resistance might have gone undetected. This constitutes a minor limitation of the study.

### Microbiological therapy

Septic lesions were defined by clinical signs of infection (erythema, warmth, pus) plus a microbial load ≥10^5^ CFU/mL on culture. Cases with positive cultures but no signs of inflammation were not classified as septic but colonized. Patients with VLUs and DFUs were treated with antibiotics because a significant proportion (60%) had septic ulcerations, confirmed by clinical assessment and microbiological swab cultures. These infected wounds showed colonization with pathogenic bacteria, including *Klebsiella pneumoniae*, *Pseudomonas aeruginosa*, and *Escherichia coli*. The clinical indications for antibiotic therapy were standard: presence of infection signs such as purulent exudate, surrounding cellulitis, malodor, pain, and positive bacterial cultures exceeding 10^5^ CFU/mL, which are thresholds conventionally associated with clinically significant infection rather than mere colonization. Patients received either a single antibiotic regimen or multiple antibiotic courses before starting ozone therapy, with the antibiotics including amoxicillin-clavulanate, cephalexin, clindamycin, ceftriaxone, and daptomycin. These choices reflect coverage for common pathogens encountered in skin and soft tissue infections, particularly in compromised hosts such as diabetics or those with chronic venous insufficiency. As for the determination of the absence of antibiotic-resistant bacteria, the manuscript indicates that none of the patients exhibited resistant infections at baseline, but the description lacks methodological detail. From the available text, it appears that standard microbiological cultures were performed using McConkey agar (and mannitol salt agar or blood agar where appropriate) to isolate and identify pathogens. However, no specific mention is made of antimicrobial susceptibility testing (AST)—such as Kirby-Bauer disk diffusion, automated systems like VITEK, or broth microdilution—being performed to assess resistance profiles explicitly. In proper practice, determining the absence of resistant organisms requires AST to be conducted on all positive cultures according to Clinical and Laboratory Standards Institute (CLSI) guidelines or equivalent protocols. These tests would determine minimum inhibitory concentrations (MICs) and identify resistance patterns to first-line and second-line antibiotics. Without explicit reporting of such testing, the assertion that no resistant bacteria were found must be considered incomplete and potentially overstated. Thus, while the study reasonably justifies antibiotic treatment based on clinical infection and microbiological positivity, it would be scientifically stronger if the authors clarified that antimicrobial susceptibility testing was systematically conducted, and detailed whether resistance was assessed against multiple antibiotic classes. If such tests were not done, this represents a limitation that should be acknowledged in the manuscript. Moreover, since diabetic and venous ulcers frequently harbor multidrug-resistant organisms—especially in recurrent or chronic cases — the absence of resistant bacteria is somewhat surprising and should be better contextualized, either as a true finding supported by testing or a limitation of incomplete microbiological assessment.

### Pain Numeric Rating Scale questionnaire

Evaluation of pain scoring was done according to a NRS questionnaire and following recent recommendations.^34^ Data was elaborated with a STATA v.18.0 software for statistics with Kruskal-Wallis test (*P* < .05).

## Oxygen-ozone therapy according to SIOOT protocols

A Multioxygen Medical 95 (Bergamo) instrumentation was used to release ozone in a standardized mixture with oxygen. The device, which is only for medical use, allows the operator to easily customize the gas mixture according to the therapy protocol. The generation by corona discharge of the O_2_-O_3_ mixture, containing 5% O_3_, is tuned and managed by a microprocessor and ozone probed by UV spectrometry at 253.7 nm, ensuring a rigorous precision of the ozone delivery once the O_3_-O_2_ mixture amount is selected by the user. Therefore, the device allows customization of the treatment dose by selecting the ozone concentration in a continuous range from 1 to 100 μg of O_3_. The treatment with oxygen-ozone represented at least two different and concurrent methods:

(a) Topical treatment exerted either using ozone disposable sterile bags for 10 minutes at 10 μg/mL three times;
(b) Injection of a range from 2 to 7 μg/mL O_3_, around or inside the lesions.
(c) Major autohemotherapy (O_2_-O_3_-MAHT), with 40 to 50 μg/mL O_3_, as previously described.^27^^,^^29^

**Supplementary Figure 2:**
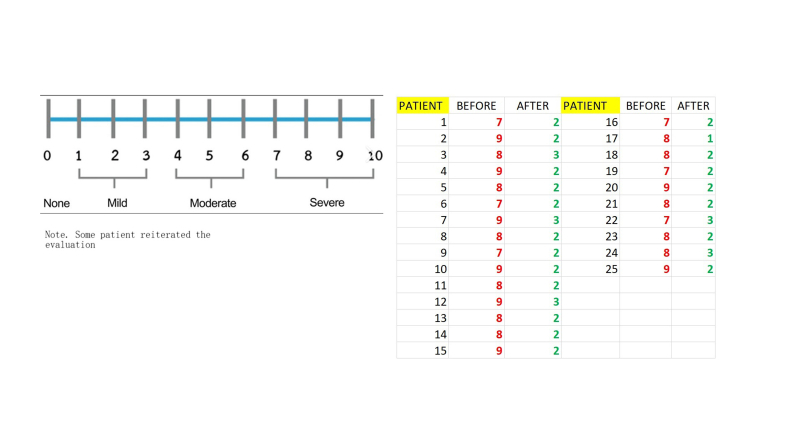
An illustration showing the oxygen-ozone major autohemotherapy.

For major autohemotherapy (MAHT), standard peripheral venous access was used in all patients, typically via the antecubital vein, using 18- to 20-gauge butterfly or intravenous catheters (Supplementary Fig S2, online only). This approach was chosen to facilitate both blood withdrawal (approximately 200 mL) and reinfusion after ozonation. Throughout the study, no major complications related to intravenous access were observed. Minor events included:

• Mild local hematoma or ecchymosis at the puncture site in three patients; and
• Mild vein irritation in two patients, which resolved spontaneously without intervention.

No cases of phlebitis, infection, thrombosis, or systemic reactions were reported.

### Protocols of oxygen-ozone therapy

Protocols followed the recommendations of the Italian SIOOT.^29^

Treatment protocols differed from septic lesions with respect to nonseptic lesions

Septic lesions (n = 15) were treated as follows: For 2 weeks:

(1) Three sessions/week with one O_2_-O_3_-MAHT (50 μg/mL O_3_) and one O_2_-O_3_-MAHT (40 μg/mL O_3_);
(2) Three routes with ozone bags as reported in (a); and
(3) Three injections as described in (b).

Then for 4 weeks:

(1) One sessions/15 days with one O_2_-O_3_-MAHT (30 μg/mL O_3_);
(2) One route with ozone bags as reported in (a) but with 15 μg/mL O_3_; and
(3) One injection as described in (b).

Then for 4 weeks:

(1) One sessions/15 days with one O_2_-O_3_-MAHT (30 μg/mL O_3_);
(2) One route with ozone bags as reported in (a); and
(3) One injection as described in (b).

Before starting OOT, surgical debridement was performed. Finally, patients were maintained with proper pharmacology and compression.

For patients with non-septic lesions, treatment included 2 weeks of:

(1) Two sessions/week with one O_2_-O_3_-MAHT (30 μg/mL O_3_);
(2) Two routes with ozone bags as reported in (a); and
(3) Two injections as described in (b).

Then for 4 weeks:

(1) One session/week with one O_2_-O_3_-MAHT (30 μg/mL O_3_);
(2) Two routes with ozone bags as reported in (a); and
(3) Two injections as described in (b).

Then for 4 weeks:

(1) One session/15 days with by one O_2_-O_3_-MAHT (30 μg/mL O_3_);
(2) One route with ozone bags as reported in (a); and
(3) One injection as described in (b).

Before starting OOT, surgical debridement was performed. Finally, patients were maintained with proper pharmacology and compression.

### Statistics

The sample size was calculated to achieve a 95% confidence level with a margin of error less than 5%, specifically targeting an error threshold of ±4.76%. The primary outcome measure was the proportion of patients achieving complete wound healing at the end of the 12-week treatment period. The calculation followed standard statistical methods for estimating a proportion in a population, using the formula:

$$n=\frac{Z^{2}\cdot \;p\;\cdot \;\left(1-p\right)\;}{E^{2}}$$

where n = required sample size, Z = z-score corresponding to the desired confidence level (1.96 for 95%), p = estimated proportion of success (assumed to be 0.5 to maximize sample size conservatively), and E = margin of error (0.0476). Using these parameters, the estimated minimum required sample size was within 25 patients.

Briefly, sample size calculation was based on the expected wound healing rate improvement from 60% (conventional care) to 85% with OOT. Using a two-sided test at α = .05 and power = 0.8, a minimum of 24 patients, as reported, was needed.

To account for potential dropouts and to further ensure robustness, 25 patients were recruited. This approach guarantees that the observed proportion of complete wound healing can be estimated with high precision, minimizing type I and type II errors in the interpretation of therapeutic efficacy. The rationale follows established biostatistical methods for binary outcome evaluation.^34^

Data were reported as mean ± standard deviation; scoring ordinal values were evaluated with a Kruskal-Wallis test for *P* < .05. A logistic regression was evaluated following previously reported methods^34^ to evaluate the rate of success/failures upon OOT. STATA v. 18.0 and SPSS v 24.0 were used for calculations. In this evaluation, “success” was defined as the complete epithelialization of the ulcer with no signs of infection or exudate by week 10, confirmed by clinical examination and a negative microbiological swab (bacterial load <10^4^ CFU/mL). Correspondingly, “failure” was defined as the persistence of open lesions, signs of infection, or lack of significant reduction in wound area (>25%) by the end of treatment.

## Results

Two patients withdrew from the study, citing improvement of symptoms before protocol completion. Thus, 25 patients completed the full treatment protocol. The cohort included 18 patients with VLUs and seven with DFUs. Of these, vascular assessment confirmed preserved arterial circulation (ABI between 0.7 and 1.3) in all patients, and no participant exhibited critical limb ischemia or required revascularization before entering the study. Moderate arterial calcifications were present in six patients but did not impact perfusion adequacy.

Venous duplex assessment revealed reflux in 16 patients: 10 cases involved superficial venous reflux, and six cases involved deep venous reflux. In the superficial group, eight patients underwent prior endovenous ablation, whereas two were managed with optimized compression therapy. Patients with deep venous reflux received conservative compression-based management. All vascular assessments and necessary interventions were completed before initiation of ozone therapy.

At 4 weeks after starting OOT, all patients exhibited fundamental improvements in their lower limbs, which improved their health status, wellness, and trust in the possibility of escaping from their concerns about undergoing limb amputation and resuming a healthy life.

Biochemical inflammatory markers at time 0 (ESR, 45.34 ± 11.22 mmHg; CRP, 35.21 ± 10.43 mg/L; and IL-6, 40.32 ± 15.67 pg/mL), improved notably at 4 weeks (ESR, 25.11 ± 10.78 mmHg; CRP, 11.37 ± 5.88 mg/L; and IL-6, 15.63 ± 7.77 pg/mL), and normalized following 10 weeks (ESR, 13.22 ± 8.34 mmHg; CRP <5 mg/L; and IL-6 <7 pg/mL).

**Fig 1:**
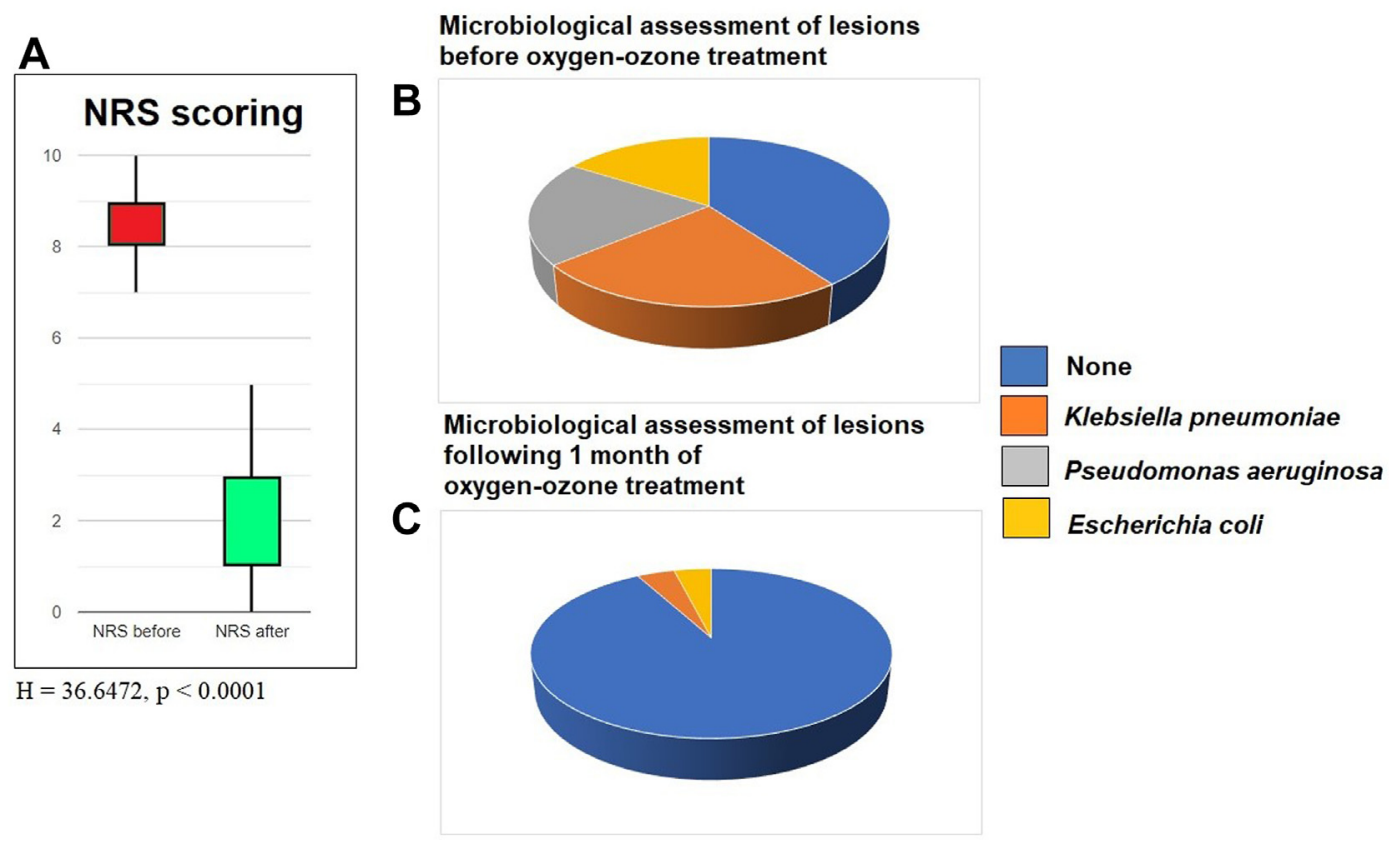
**(A)** Numeric Rating Scale (*NRS*) score values before oxygen-ozone therapy (OOT) and following the whole cycle of OOT, according to the Methods described in the text. **(B)** Microbiological assessment (McConkey agar test) of lesions before OOT. None: 10 cases; *K. pneumoniae* = 6 cases; *P. aeruginosa* = 5 cases; *E. coli* = 4 cases. **(C)** Microbiological assessment (McConkey agar test) of lesions following 1 month of OOT. None = 23 cases; *K. pneumoniae* = 1 case; *P. aeruginosa* = 0 cases; *E. coli* = 1 case. Microbiological tests in Mc Conkey agar.

Therefore, patients exhibited substantial clinical improvements. Pain scores, assessed by the NRS, were 8.08 ± 1.25 before OOT and 2.16 ± 0.50 following OOT, dropping pain sensitivity about 73.27% at the first month of OOT treatment. Kruskal-Wallis test for score values reported that this difference was significant at *P* < .0001 (H = 36.6472) (Fig 1, *A*). Evaluation was performed with two independent interviewers, giving a Cohen’s k = 0.6725, which, according to Landis and Koch’s estimation, should represent a good accordance. Two blinded independent reviewers assessed pain scoring (NRS) at baseline and end of study. Cohen’s kappa was calculated to assess inter-rater reliability.

Bacterial colonization before OOT included 15 patients with septic ulcerations ([60%] with 24% *Klebsiella pneumoniae*, 20% *Pseudomonas aeruginosa*, and 16% *Escherichia coli*) (Fig 1, *B*), whereas following OOT, negative cutaneous swabs (CFU/mL ≤ 10^4^) amounted to 92% (Fig 1, *C*). An evident reduction of the inflammatory biomarkers was also reported.

**Fig 2:**
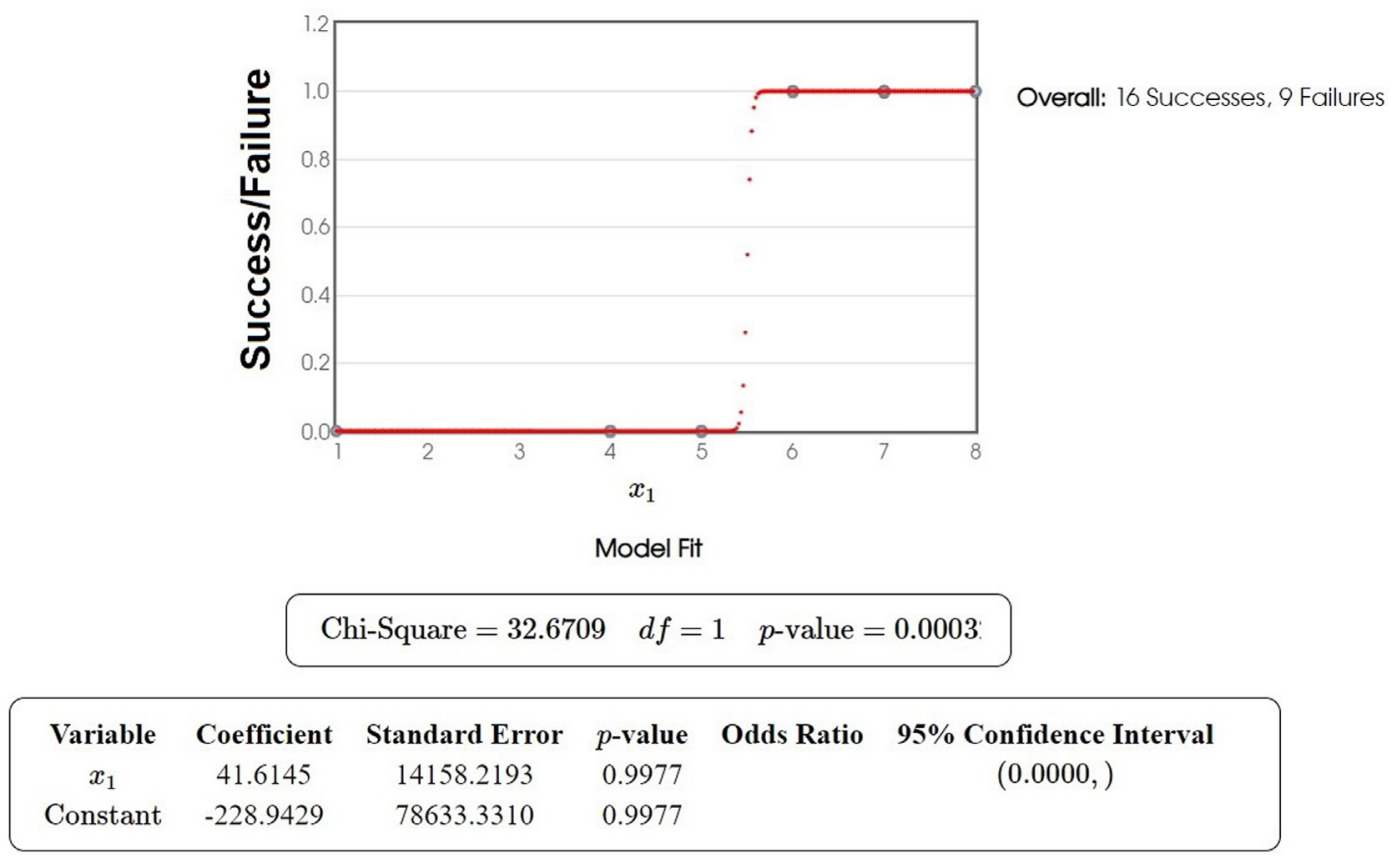
Logistic regression of success/failure according the Cox's method used to analyze datasets in which one or more independent variables determine a binary outcome upon oxygen-ozone therapy (OOT) treatments. The outcome variable is categorical and often represents a dichotomous result such as success/failure or 0/1. Logistic regression models the probability of the default class using the logistic function, ensuring the predicted probabilities lie within the interval [0,1] (ie, failure, success).

Logistic regression resulted in 16 successes and nine failures (P < .0001; χ2 = 32.6709) (ie, that OOT quite doubles the possibility of successes due only to chance), clearly assessing an effect of OOT on the VLUs investigated in the study (Fig 2). Success was defined as complete epithelialization of the ulcer with no signs of infection or exudate by week 10, confirmed by both clinical inspection and negative microbiological swabs (CFU < 10^4^/mL); B) Failure was defined as the persistence of open lesions, presence of infection, or lack of significant wound reduction (>25% area) by the end of treatment. Importantly, none of the patients in the study required limb amputation during or after the study period. This outcome supports the potential of OOT as a limb-sparing intervention for advanced VLUs and DFUs.

**Fig 3:**
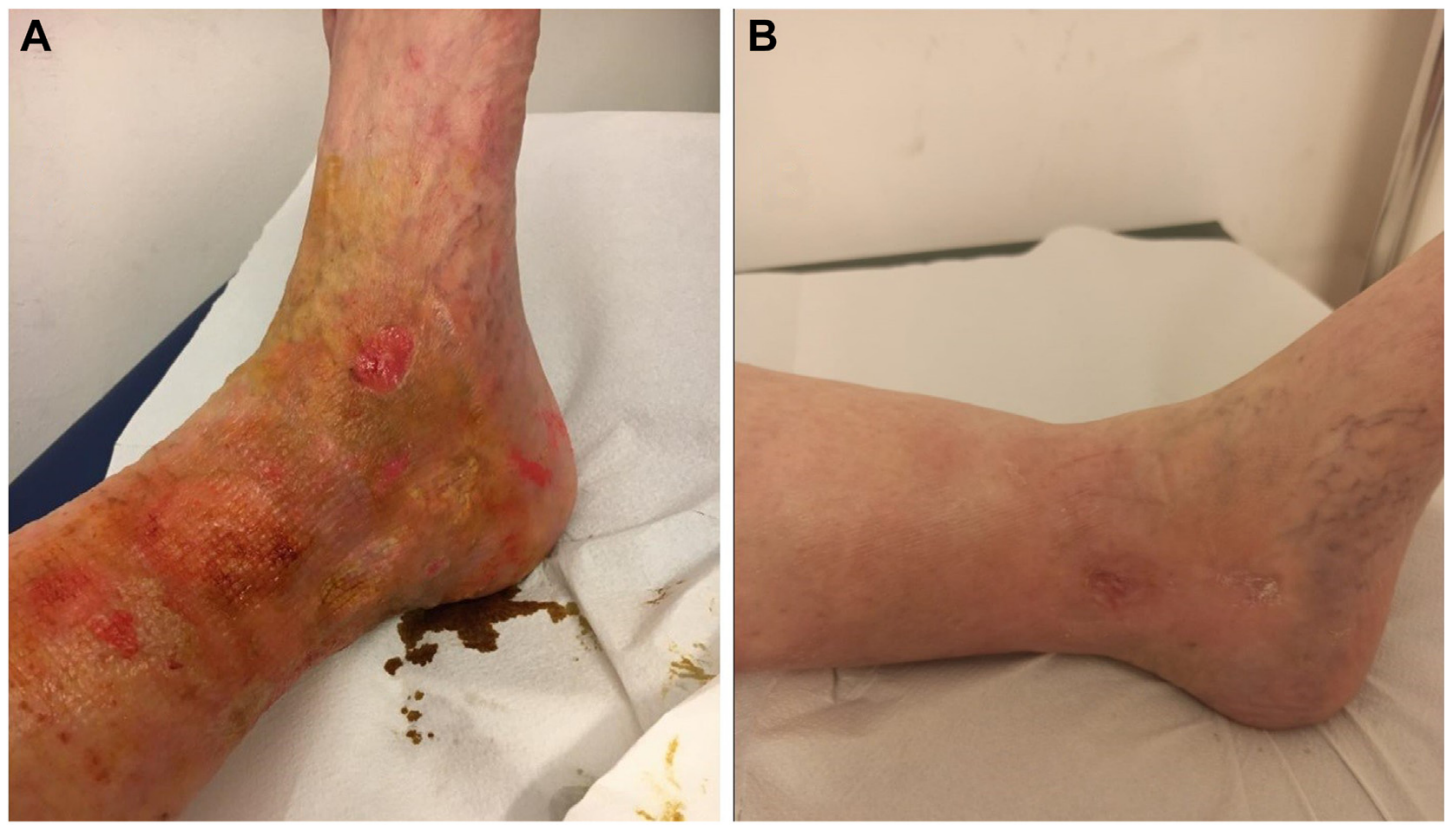
Outcome of oxygen-ozone therapy (OOT) before **(A)** and following 5 sessions of OOT **(B)** in a 65-old patient with venous leg ulcer (VLU).

**Fig 4:**
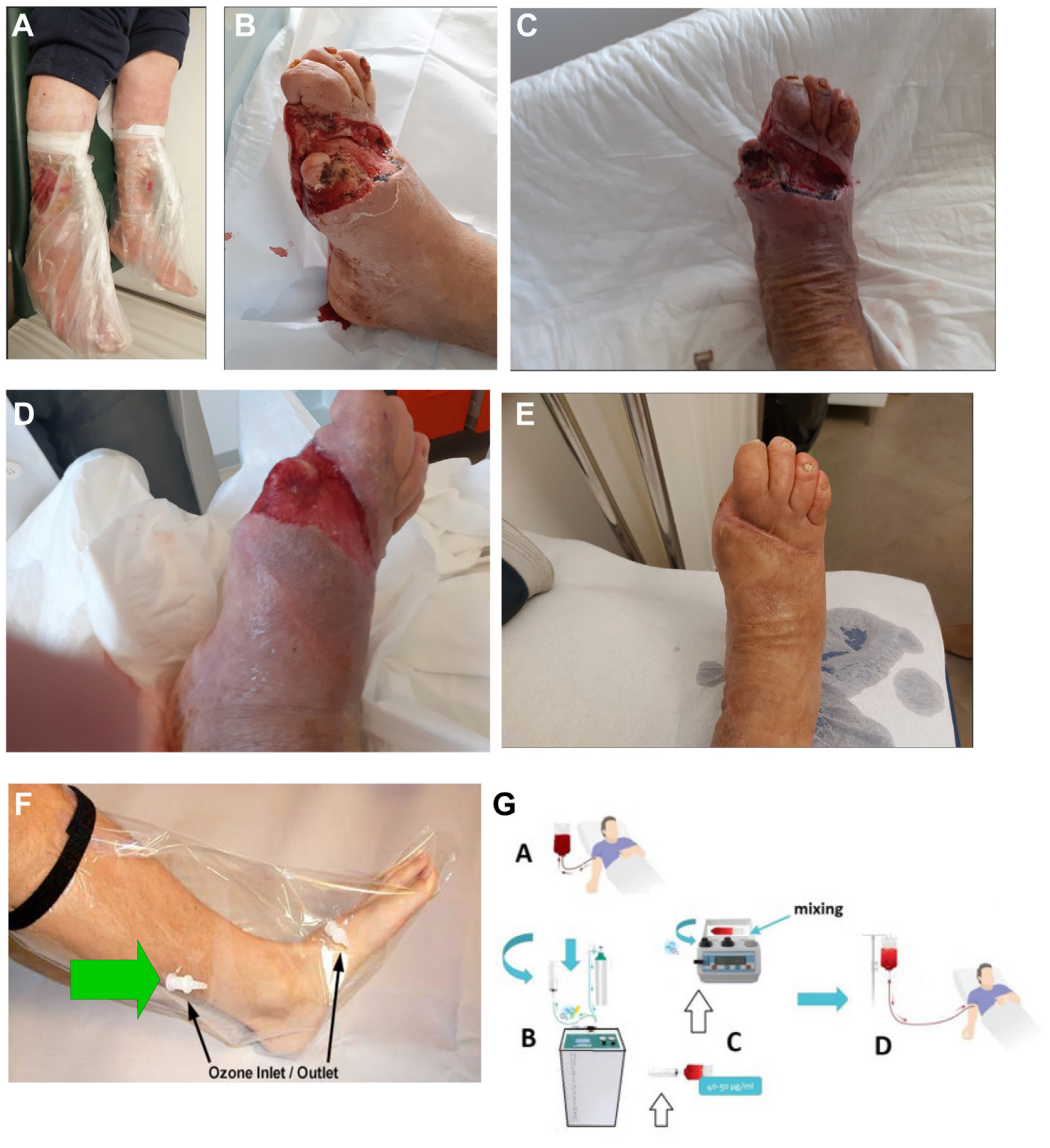
**(A)** General methodology with ozone bags used for the study. Panels from **(B)** to **(E)** illustrate progressive healing following oxygen-ozone therapy (OOT), according to the Italian Scientific Society of Oxygen-Ozone Therapy (SIOOT) protocol described in methods, of diabetic foot ulcers (DFUs) in a 71-year-old male patient. **(B)** Before treatment; **(C)** following 4 sessions of OOT; **(D)** following 10 sessions of OOT; **(E)** follow-up at 2 months following OOT. The patients were initially scheduled for limb amputation. **(F)** Methods of OOT in the bag. In a typical ozone therapy bag used for treating wounds (often called a “topical ozone bag” or “ozone limb bag”), the setup involves two ports: **(A)** Inlet port (disposable inlet tube): this is the entry point for the medical ozone-oxygen gas mixture. It is connected to the ozone generator using ozone-resistant tubing (typically silicone or Teflon). The inlet is often fitted with a one-way valve or luer lock connector to prevent gas backflow and ensure safe delivery of the mixture into the bag. **(B)** Outlet port (disposable outlet or vent tube): this is the exit point for excess ozone gas after it circulates within the bag. It can be connected to an ozone destructor or neutralizer (such as one with activated carbon) to safely absorb and neutralize residual ozone. This outlet ensures there is no pressure build-up and maintains proper gas flow inside the bag. These ports are usually color-coded or clearly marked, and disposable components should be replaced between patients to prevent cross-contamination. The ozone bag is sealed around the limb with a cuff or adhesive to create an airtight chamber. **(G)** Major autohemotherapy. (1) Blood is drawn from the patient (200 mL) into a sterile disposable SANO_3_ bag; (2) Ozone is generated at protocol-specified concentrations using specialized equipment that produces ozone in a pure oxygen atmosphere (95% oxygen – 5% ozone); (3) The ozone is introduced into the bag through a designated inlet, and the bag is gently mixed; (4) The ozonated blood is then reinfused into the patient.

The effective number of patients reaching the complete ending of the study was limited to 20 patients. Fig 3 shows an exemplificative case of a patient completely recovering his health lower limb from a severe ulcerous wound (Fig 3, *A*) to a complete restitutio ad integrum (Fig 3, *B*) following a number of six OOT sessions. Fig 4 reports a severe case of diabetic foot with loss of fingers. Fig 4, *A* shows an example (not the same patient detailed in Fig 4) of using sterile ozone bags, as indicated in the Methods. Fig 4, *B* shows the patient’s feet at time 0 (before OOT), Fig 4, *C* shows the patient’s feet at 4 weeks following OOT, Fig 4, *D* shows the same patient at 10 weeks, and Fig 4, *E* shows the outcome at the end of OOT following 2 months follow-up, assessing the evolutionary progress of a 71-year-old male patient’s diabetic foot following a whole cycle of 10 OOT treatments. Fig 4, *F* details the ozone bag methodology used to treat patients’ ulcerous wounds, and Fig 4, *G* is a schematic summary of the OOT as blood autohemotherapy (O2-O3-MAHT).

## Discussion

The role of ozone in allowing VLUs to reach their restitutio ad integrum, removing sepsis whenever present and remodeling tissues and reconstruction, may leave readers quite dumbfounded. Actually, it is possible to envisage that ozone contributes in accelerating wound healing along with other pharmaceutical drugs, yet the data presented here, for which we reported only exemplificative cases, due to space constraints, clearly showed a quite complete healing of the severe damage due to VLUs and led to a better healing of DFUs, which usually are not commonly observed with conventional pharmacology alone.^35^ Moreover, despite the robust microbiological control achieved with OOT, a limitation of this study is the partial performance of antimicrobial susceptibility testing. Although no clinical signs of resistant infections were observed, systematic AST for all isolates would have provided stronger microbiological validation.

The use of oxygen-ozone to address VLUs and DFUs, sometimes along with hyperbaric oxygen, is increasing its impact in the scientific literature.^36^, ^37^, ^38^, ^39^, ^40^ A possible reason can be associated with the ozone ability to cleanse microbial contamination and sepsis from open lesions characterizing VLUs and DFUs, therefore allowing immunity to overcome any bacterial challenging, along with a correct antibiotic panel.^27^^,^^29^ Notwithstanding, in a non-septic condition, ozone might exert a powerful anti-oxidant and anti-inflammatory action, leading to the activation of the Nrf2/Keap1/ARE pathway, the expression and synthesis of HO-1, with production of CO and upregulation of the biomarker MARCO, which enhances the phagocytosis ability of these innate cells.^41^, ^42^, ^43^ Interestingly, whereas the effect of ozone therapy on tissues and limbs circulation is quite complete, the reduction of pain occurs by about 70%, approaching previous data with chronic inflammatory and painful pathologies where ozone exerted its effectiveness.^44^ Venous lower limb ulcers can be dangerous for health if not properly treated. These ulcers result from chronic venous insufficiency, where veins in the legs are unable to efficiently return blood to the heart, leading to increased pressure in the veins and damage to the skin. Several concerning issues are associated with lesions due to VLUs or DFUs. It is widely known that **o**pen ulcers are susceptible to bacterial infection, which can lead to infection of the deeper layers of skin or even more severe systemic infections like sepsis if left untreated. Moreover, venous ulcers can be slow to heal, leading to chronic wounds that persist for months or even years. This can cause ongoing pain, discomfort, and reduced quality of life. Even after healing, venous ulcers have a high recurrence rate unless the underlying venous insufficiency is managed. Again, persistent ulcers can cause significant pain and discomfort, impacting daily activities and overall well-being. Therefore, the most correct therapeutic approach is of utmost importance, if considering that chronic venous insufficiency and recurrent ulcers can lead to long-term skin changes, including lipo-dermatosclerosis (thickening and hardening of the skin) and “atrophie blanche” (white, scar-like areas). Finally, in severe cases where ulcers become critically infected or fail to heal, there is a risk of limb amputation, although this is relatively rare.

### Limitations of the study

This study provides promising results regarding the efficacy of OOT in treating VLUs and DFUs, yet it also bears several limitations that must be acknowledged. First, the sample size was relatively small, with only 25 patients completing the protocol, which may affect the generalizability of the findings. The absence of a randomized control group limits the ability to attribute observed improvements solely to OOT, as the study design cannot rule out placebo effects or natural healing progression. Furthermore, the inclusion of both VLUs and DFUs, despite their distinct etiologies, may confound interpretation of efficacy across these ulcer types. Another limitation is the selective and incomplete use of AST. Although the study claims no resistant bacteria were observed, the lack of systematic AST for all isolates leaves room for undetected resistance, potentially overstating the antimicrobial effectiveness of OOT. Additionally, the study did not systematically document ulcer area reduction in all patients, relying instead on qualitative assessments and measurements from a subgroup, which may introduce bias or reduce reproducibility. The reliance on photographic documentation, although standardized, can still be subject to interpretation. Moreover, the lack of long-term follow-up prevents conclusions about the durability of healing and recurrence rates. The concurrent use of ongoing pharmacologic therapies and individualized treatment protocols also introduces variability that complicates attribution of outcomes to OOT alone. Finally, ethical considerations such as the absence of formal ethical committee approval, although deemed non-mandatory, may limit acceptance of the findings in some research contexts. Together, these limitations highlight the need for larger, randomized, and controlled trials with standardized protocols and comprehensive microbiological assessments to validate the observed benefits of OOT in chronic wound management.

## Conclusions

Proper management of venous ulcers typically involves addressing both the wound itself and the underlying venous insufficiency. This can include compression therapy, wound care, medications, lifestyle changes, and sometimes surgical interventions. Early and consistent treatment is crucial to prevent complications and promote healing. Following OOT, no patient underwent limb amputation. In this manuscript, we have clearly defined success and failure as clinical endpoints: (1) Success was defined as complete epithelialization of the ulcer with no signs of infection or exudate by week 10, confirmed by both clinical inspection and negative microbiological swabs (CFU <10^4^/mL); (2) Failure was defined as the persistence of open lesions, presence of infection, or lack of significant wound reduction (>25% area) by the end of treatment. Importantly, none of the patients in the study required limb amputation during or after the study period. This outcome supports the potential of OOT as a limb-sparing intervention for advanced VLUs and DFUs.

## Author contributions

Conception and design: GM, SC

Analysis and interpretation: MF, UT, LV, FV, SC

Data collection: GM, FV, SC

Writing the article: SC

Critical revision of the article: GM, MF, UT, LV, FV, SC

Final approval of the article: GM, MF, UT, LV, FV, SC

Statistical analysis: MF, SC

Obtained funding: Not applicable

Overall responsibility: SC

## Funding

None.

## Disclosures

None.
